# A step forward toward solving the main mysteries in the history of plague?

**DOI:** 10.1073/pnas.2221925120

**Published:** 2023-03-06

**Authors:** Guido Alfani

**Affiliations:** ^a^Bocconi University–Dondena Centre for Research on Social Dynamics and Innocenzo Gasparini Institute for Economic Research, 20136 Milan, Italy

Stenseth et al. (1) claim that local environmental factors in Western and Central Europe, including the characteristics of rodent and ectoparasite communities and a range of climatic and morphological factors, do not support the view that this area hosted long-term plague reservoirs. From a historical point of view, this claim appears reasonable and contributes to the debate about plague persistence in Europe vs continuous reintroduction (2). However, Stenseth et al. fail to recognize other, broader implications of their results.

First, there is the mystery of the disappearance of the plague from Europe. Scholars have highlighted various factors which might have played a role, alone or in combination. These include environmental factors (variations in the population of vectors of the disease, perhaps associated with climate change, or mutual adaptation between humans and pathogens), epidemiological factors (the exceptional severity of the last great plagues of the seventeenth century, possibly reflecting pathogen mutation, led the infection to extinguish itself) as well as institutional factors (improvements in public health and in hygiene) (3, 4). As a result, from the turn of the 18th century, plague circulation in Western and Central Europe was very limited compared to the broader Mediterranean area and Asia. Some episodes, such as the 1720 to 1722 plague in Marseille and that of 1820 to 1822 in Mallorca, were due to reinfection from the outside through naval trade. While locally serious, they remained territorially circumscribed. Stenseth et al.’s findings do not provide a direct solution to the mystery. However, they do point at conditions that might have made it relatively easy for Europe to get rid of plague and in this sense, they make a substantial contribution toward finding, one day, the solution.

Stenseth et al.’s findings are also relevant to another plague mystery: How was *Yersinia pestis* able to cause the greatest mortality crises ever reported in European history (Table 1)? An infection that requires different kinds of vectors (rats, fleas) to spread does not reconcile very well with the quick diffusion across Europe of the Black Death and other major plagues (5, 6). In the past, attempts at squaring the circle introduced in epidemiological models ad hoc hypotheses about the prevalence and typology of rodents. They assumed that past plagues must have spread exactly as observed in modern outbreaks, for example, in India—which is not the case (6, 7). Stenseth et al. claim that European rodent and ectoparasite communities were probably not very suitable for the sustained survival of *Y. pestis* in Western and Central Europe. This implicitly supports what many have long suspected: In the specific environmental conditions of the main European plagues, human-to-human transmission (including through ectoparasites, maybe including lice beyond fleas, but without involving rodents) must have been easier than observed in modern epidemics (4, 7, 8). This finding is compatible with recent microdemographic studies of 17th-century plagues (9, 10). A tentative estimate is that about three-quarters of plague infections occurred human-to-human (10).

**Table 1.** Main plague outbreaks affecting Europe and the Mediterranean

	Regions affected	Victims (millions)	Mortality rate (% of population killed)
540 to 541 (possibly up to ca. 550 in northern Europe)—Justinianic Plague	Europe, Mediterranean	Up to 25 to 50 overall	25 to 50% overall (50% in Egypt and other densely populated areas)
1346 to 1352—Black Death	Europe, Mediterranean, Middle East, central Asia, possibly parts of China and other areas	Up to 50 in Europe and the Mediterranean; unknown elsewhere	35 to 60% in Europe and the Mediterranean; unknown elsewhere
1356 to 1366—*pestis secunda*	Europe, Mediterranean, Middle East	Up to 5 to 10 in Europe and the Mediterranean; unknown elsewhere	15 to 20% in Europe and the Mediterranean; unknown elsewhere
1625 to 1632	Most of central and western Europe (areas spared include most of Spain and central-southern Italy)	Up to 2 in northern Italy; up to 1.15 in France; up to 0.25 in Switzerland; up to 0.16 in the Dutch Republic; unknown elsewhere	30 to 35% in northern Italy, 20 to 25% in Switzerland; 20 to 25% in South Germany, Rhineland and Alsace (up to 40% if also victims of famines and of the Thirty Years’ War are included); 8 to 11% in the Dutch Republic; unknown elsewhere
1647 to 1657	Andalusia, Spanish Mediterranean, and central-southern Italy	Up to 1.25 in the Kingdom of Naples; up to 0.5 in Spain; up to 0.33 in France; unknown elsewhere	30 to 43% in the Kingdom of Naples; at least 25% in Andalusia; 15 to 20% in Catalonia; unknown elsewhere

Future plague studies based on biological evidence would do well to pay greater attention to findings from historical research or risk missing the main questions.
